# The power of peers: an effectiveness evaluation of a cluster-controlled trial of group antenatal care in rural Nepal

**DOI:** 10.1186/s12978-019-0820-8

**Published:** 2019-10-22

**Authors:** Poshan Thapa, Alex Harsha Bangura, Isha Nirola, David Citrin, Bishal Belbase, Bhawana Bogati, B. K. Nirmala, Sonu Khadka, Lal Kunwar, Scott Halliday, Nandini Choudhury, Al Ozonoff, Jasmine Tenpa, Ryan Schwarz, Mukesh Adhikari, S. P. Kalaunee, Sharon Rising, Duncan Maru, Sheela Maru

**Affiliations:** 1 University of New South Wales, School of Public Health and Community Medicine, Sydney, NSW Australia; 2 Lakewood Health System, Staples, MN USA; 3 Harvard T.H. Chan School of Public Health, Boston, MA USA; 4 Nyaya Health Nepal, Kathmandu, Nepal; 5 Department of Anthropology, University of Washington, Seattle, WA USA; 6 Department of Global Health, University of Washington, Seattle, WA USA; 7 University of Washington, Henry M. Jackson School of International Studies, Seattle, WA USA; 8 Icahn School of Medicine at Mount Sinai, Arnhold Institute for Global Health, 1216 Fifth Avenue, Fifth Floor, Room 556, New York, NY 10029 USA; 9 Karma Health, Kathmandu, Nepal; 10 Om Health Science Campus, Purbanchal University, Kathmandu, Nepal; 11 Center for Patient Safety and Quality Research, Boston Children’ Hospital, Boston, MA USA; 12 Department of Medicine, Harvard Medical School, Boston, MA USA; 13 Department of Medical Oncology, Sidney Kimmel Cancer Center, Thomas Jefferson University, Philadelphia, PA USA; 14 Department of Medicine, Division of Global Health Equity, Brigham and Women’s Hospital, Boston, MA USA; 15 Department of Medicine, Division of General Internal Medicine, Massachusetts General Hospital, Boston, MA USA; 16 Yale School of Public Health, New Haven, CT USA; 17 Eastern University, College of Leadership and Development, St. Davids, PA USA; 18 Group Care Global, Silver Spring, MD USA; 19 Department of Health Systems Design and Global Health, Icahn School of Medicine at Mount Sinai, New York, NY USA; 20 Department of Internal Medicine, Icahn School of Medicine at Mount Sinai, New York, NY USA; 21 Department of Pediatrics, Icahn School of Medicine at Mount Sinai, New York, NY USA; 22 Department of Obstetrics, Gynecology and Reproductive Science, Icahn School of Medicine at Mount Sinai, New York, NY USA

**Keywords:** Centering pregnancy, Child health, Group antenatal care, Implementation research, Maternal health, Peer group, Prenatal care

## Abstract

**Background:**

Reducing the maternal mortality ratio to less than 70 per 100,000 live births globally is one of the Sustainable Development Goals. Approximately 830 women die from pregnancy- or childbirth-related complications every day. Almost 99% of these deaths occur in developing countries. Increasing antenatal care quality and completion, and institutional delivery are key strategies to reduce maternal mortality, however there are many implementation challenges in rural and resource-limited settings. In Nepal, 43% of deliveries do not take place in an institution and 31% of women have insufficient antenatal care. Context-specific and evidence-based strategies are needed to improve antenatal care completion and institutional birth. We present an assessment of effectiveness outcomes for an adaptation of a group antenatal care model delivered by community health workers and midwives in close collaboration with government staff in rural Nepal.

**Methods:**

The study was conducted in Achham, Nepal, via a public private partnership between the Nepali non-profit, Nyaya Health Nepal, and the Ministry of Health and Population, with financial and technical assistance from the American non-profit, *Possible*. We implemented group antenatal care as a prospective non-randomized, cluster-controlled, type I hybrid effectiveness-implementation study in six village clusters. The implementation approach allowed for iterative improvement in design by making changes to improve the quality of the intervention. We evaluated effectiveness through a difference in difference analysis of institutional birth rates between groups prior to implementation of the intervention and 1 year after implementation. Additionally, we assessed the change in knowledge of key danger signs and the acceptability of the group model compared with individual visits in a nested cohort of women receiving home visit care and home visit care plus group antenatal care. Using a directed content and thematic approach, we analyzed qualitative interviews to identify major themes related to implementation.

**Results:**

At baseline, there were 457 recently-delivered women in the six village clusters receiving home visit care and 214 in the seven village clusters receiving home visit care plus group antenatal care. At endline, there were 336 and 201, respectively. The difference in difference analysis did not show a significant change in institutional birth rates nor antenatal care visit completion rates between the groups. There was, however, a significant increase in both institutional birth and antenatal care completion in each group from baseline to endline. We enrolled a nested cohort of 52 participants receiving home visit care and 62 participants receiving home visit care plus group antenatal care. There was high acceptability of the group antenatal care intervention and home visit care, with no significant differences between groups. A significantly higher percentage of women who participated in group antenatal care found their visits to be ‘very enjoyable’ (83.9% vs 59.6%, *p* = 0.0056). In the nested cohort, knowledge of key danger signs during pregnancy significantly improved from baseline to endline in the intervention clusters only (2 to 31%, *p* < 0.001), while knowledge of key danger signs related to labor and childbirth, the postpartum period, and the newborn did not in either intervention or control groups. Qualitative analysis revealed that women found that the groups provided an opportunity for learning and discussion, and the groups were a source of social support and empowerment. They also reported an improvement in services available at their village clinic. Providers noted the importance of the community health workers in identifying pregnant women in the community and linking them to the village clinics. Challenges in birth planning were brought up by both participants and providers.

**Conclusion:**

While there was no significant change in institutional birth and antenatal care completion at the population level between groups, there was an increase of these outcomes in both groups. This may be secondary to the primary importance of community health worker involvement in both of these groups. Knowledge of key pregnancy danger signs was significantly improved in the home visit plus group antenatal care cohort compared with the home visit care only group. This initial study of Nyaya Health Nepal’s adapted group care model demonstrates the potential for impacting women’s antenatal care experience and should be studied over a longer period as an intervention embedded within a community health worker program.

**Trial registration:**

ClinicalTrials.gov Identifier: NCT02330887, registered 01/05/2015, retroactively registered.

## Plain English summary

About 830 women die during pregnancy or childbirth every day, most in low- and middle-income countries. Encouraging women to give birth in safe medical facilities is a key strategy to reduce these deaths, however this is not easy to do. We started a group pregnancy care program in rural Nepal towards this effort. Women receive pregnancy care in groups, increasing time with healthcare providers, quality of care, and social support.

We studied the program to see if it was helpful in increasing the proportion of women giving birth in medical facilities. We looked at two sets of villages, one set where we ran the group pregnancy program, and another set where we did not. We looked at both sets of villages before the start of the program and 1 year later, and we compared any changes we observed. We also asked some women in each set of villages about their knowledge about pregnancy and their satisfaction with pregnancy care, before and after the program.

We found that adding group pregnancy care did not increase the proportion of women delivering in a medical facility. However, women who had group pregnancy care found it more enjoyable and had better pregnancy knowledge. In interviews, women said that groups provided learning, discussion, social support and empowerment. While we cannot say that adding group care will promote women giving birth in a medical facility, this initial study shows the potential for impacting women’s pregnancy care experience.

## Background

Maternal mortality continues to devastate communities in much of the world: approximately 830 women die every day due to complications related to pregnancy and childbirth [1]. Almost 99% of these deaths occur in low- and middle-income countries (LMICs), with about one-third concentrated in South Asia [1]. Reducing the maternal mortality ratio to less than 70 deaths per 100,000 live births in every country is one of the Sustainable Development Goals [2]. Nepal is one of South Asia’s most impoverished countries and presents a challenging environment in which to achieve maternal mortality goals. Global evidence has shown that quality antenatal care (ANC) and institutional delivery services are key strategies to reduce maternal mortality [3, 4], yet they are often lacking or severely underutilized [5, 6].

Nepal has significantly reduced the maternal mortality ratio from 850 deaths per 100,000 live births in 1990 to 239 in 2016 [7, 8]. This success can be attributed to a series of national programs that addressed both supply and demand-side challenges. The National Safe Motherhood Program was initiated in 1997, a comprehensive 15 year strategy which included expanded emergency obstetric service access [9]. To promote ANC utilization and institutional delivery, the Ministry of Health and Population introduced the Safe Motherhood Program involving a maternal cash incentive scheme in 2005. In 2009, user fees were removed from all types of maternity care in public sector healthcare facilities [10]. Additionally, the government has implemented programs around rural ultrasound, emergency referral trainings, and post-partum hemorrhage management, among other programs. The most recent Demographic and Health Survey reports that 57% of deliveries in Nepal are institutional and 69% of woman complete four or more ANC visits [8]. In order for Nepal to achieve the Sustainable Development Goal targets, context-specific and evidence-based strategies are needed to further improve ANC completion and institutional birth.

Nyaya Health Nepal, a Nepali healthcare organization operates in partnership with the Ministry of Health and Population and the American non-profit *Possible* to strengthen integrated care delivery systems. This partnership has been working towards improving access to ANC and institutional birth services through operating the government-owned Bayalpata Hospital and a community health worker (CHW) network in far-western Nepal since 2008. As described in the accompanying piece (under review by Reproductive Health [11]), our team has designed an innovative group ANC model adapted from the Centering Pregnancy model in order to promote women’s empowerment and social support network development to address resource gaps and sociocultural barriers to care [12]. ANC recommendations published in 2016 by the World Health Organization endorse community-based mobilization to improve access to care and outcomes, especially in rural parts of LMICs [4]. They recommend group care models as potentially beneficial for improving the quality of care and call for additional research of these models. To this end, we developed a group ANC model appropriate to the rural Nepal context.

The key aspects of the refined group ANC model are: adapted gestational-age matched groups; facilitation conducted by CHWs and a nurse-midwife; location within a village clinic (known locally in Nepal as a (sub-)health post); education; social support;, and decentralized prenatal labs and ultrasound services. Through a type 1 hybrid-effectiveness-implementation study [13], we evaluated the effect of this model on two key outcomes: the institutional birth rate and completion of four ANC visits, in addition to process indicators of knowledge acquisition and birth preparedness.

## Methods

### Study setting

This study was implemented in Sanfebagar, a rural municipality located in Province 7, a far-western hilly region of Nepal. At the time of this study Nyaya Health Nepal’s community health worker (CHW) network served a direct catchment area population of 36,000 people across 14 village clusters. In each village cluster, a village clinic provides basic outpatient primary care services and many provide birthing services. For higher-level services, the population in Sanfebagar generally utilizes Bayalpata Hospital, the district-level hospital supported by Nyaya Health Nepal. Travel times to the hospital vary between village clusters, and can be up to 4 hrs by foot. Travel times are significantly lengthened during the monsoon season, which is 3 months of the year.

### Group ANC intervention

Six of the 13 village clusters in the study population offered the group ANC program out of their local village clinics to all women presenting for antenatal care (at < 24 weeks gestation). Piloting and iteration of the group intervention was conducted in the same village clusters from September 2014 to February 2015, which is described in detail in a complementary paper [11]. The refined group ANC program was implemented beginning March 2015 and continued to present. Groups received 4 two-hour antenatal sessions facilitated by a government nurse-midwife and a Nyaya Health Nepal-employed CHW that included ANC and facilitated peer discussion on a variety of pregnancy topics and birth planning. These intervention clusters were selected by convenience, based on political and programmatic considerations. In contrast, seven (control) village clusters offered the existing standard of antenatal care, as per national guidelines (i.e. four individual patient visits at local village clinics conducted by government nurse-midwives). In all 13 village clusters, Nyaya Health Nepal additionally implemented a CHW program organized around: 1) CHW payment; 2) supervision; 3) digital support tools; 4) local recruitment to optimize the standard of care, in which trained lay healthcare workers visited all pregnant women monthly throughout their gestation. The intervention and optimized standard of care are described in detail in a complementary paper [11].

### Study design

We studied the intervention using a prospective non-randomized, cluster-controlled, type I hybrid effectiveness-implementation trial design with a nested prospective cohort. A randomized design was not feasible due to government priorities in the study population. The primary population-level outcomes of interest were the institutional birth rate and completion of at least four ANC visits, while secondary outcomes were the: (i) one-year postpartum modern contraceptive prevalence rate (PPCPR); (ii) stillbirth rate; (iii) perinatal mortality rate; and (iv) infant mortality rate. We did not include maternal mortality or morbidity outcomes due to the small sample size and data limitations from self-reported birth outcomes.

We surveyed the nested cohort to assess participants’ knowledge of birth preparedness and key danger signs during pregnancy, labor and child birth, the post-partum period, and in the newborn. The survey tool was adapted from Jhpiego’s “Monitoring birth preparedness and complication readiness: tools and indicators for maternal and newborn health” [14]. In addition, we assessed cohort participants’ attitudes and practices after delivery, including satisfaction with their ANC experience, birth planning practices, and barriers to institutional delivery. We also conducted focus group discussions (FGDs) and key informant interviews (KIIs) to explore the experience of the model and perceived mechanisms of impact, from a variety of perspectives. These qualitative data were only collected from those with direct experience with the intervention.

### Participants and sampling

#### Population-level census

We aimed to exhaustively sample two independent comparison groups across the 13 village clusters to compare population-level outcomes before and after implementation of the intervention. These consisted of all married women of reproductive age (15–49 years) in the catchment area population, who had given birth within the 2 yrs prior to the survey date. These data were collected in the catchment area population before implementation of the intervention through a household census and after a full year of implementation. The pre-implementation survey was conducted from November 2014 to February 2015. The follow-up survey was conducted March 2016 to July 2016.

#### Nested cohort

We used convenience sampling to enroll the first 6–15 women identified for ANC in each village cluster during the initial 3 months of the intervention (March through May 2015). All pregnant women in their first or second trimesters were eligible for enrollment. In intervention clusters, women were recruited at the first group ANC visit. In control clusters, recruitment took place after CHWs enrolled newly identified pregnant women in routine ANC home visits.

#### Key informants and focus group discussion

We purposively sampled key informants to maximize heterogeneity in terms of positionality and other social and status markers, such as age, sex, and caste. The group ANC participant key informants were selected to represent different birth experiences and health outcomes. Additionally, in the intervention village clusters, we conducted a focus group discussion (FGD) with each ANC group after their postnatal group session. The FGD and KII participants were provided with snacks, otherwise there were no financial incentives.

### Sample size and power calculations for population-level measures

#### Intra-class correlation of outcome

We used data from the first household census to estimate the intra-class correlation coefficient of the binary outcome institutional birth on the clustering variable village cluster. We cross-checked our estimate using four different estimation methods including one designed specifically for binary outcomes. Point estimates for intra-class correlation coefficients ranged from 0.01 to 0.03. The highest 95% upper bound across all methods was 0.09. Therefore, we ignored design effects in our power calculations. Intra-class correlation coefficient estimates for the binary variable for completion of at least four ANC visits ranged from 0.10 to 0.15.

#### Assumptions

We assumed a pre-post comparison of independent binary outcomes with roughly equal numbers (*n* = 2400; *n* = 1200 per group) of live births in both pre- and post-implementation groups. We assumed a pre-implementation rate of either 76% for institutional birth rate or 85% for the completion of at least four ANC visits, based upon previous retrospective cohort data collected after comprehensive emergency obstetric care was implemented in the Sanfebagar catchment area population [15]. Using a two-sided significance level (alpha) of 0.05, we calculated statistical power for a test based on an unpooled z-statistic approximation and calculated the minimum detectable effect with at least 80% power under our two baseline scenarios. We calculated at least 80% power to detect an absolute difference of 5% under a scenario of 76% baseline and 4% under a scenario of 85% baseline.

### Ethics approval

The study protocol was approved by the institutional review boards of the Nepal Health Research Council (133/2014), Dhulikhel Hospital-Kathmandu University Hospital (81/14), and Brigham and Women’s Hospital (2015P000058/BWH).

### Data collection

#### Population-level census

The census questionnaire was adapted from the Demographic and Health Survey and the Multiple Indicator Cluster Survey to gather data on household demographics and a retrospective birth history for all married women of reproductive age. Data on delivery location, completion of ANC visits, contraceptive use, and birth outcomes for each pregnancy in the 2 yrs preceding the survey were collected. Nyaya Health Nepal’s CHWs administered the census within their respective village clusters. The pre-implementation census was administered between November 2014 to February 2015 using SurveyCTO, an Android-based mobile phone running an Open Data Kit application [16].

Following a transition to a new data system with greater care delivery capacity, the follow-up census was conducted from March 2016 to July 2016 using CommCare, an Android-based mobile application developed in collaboration with Nyaya Health Nepal’s technology partner Dimagi [17]. The questionnaire administered in the follow-up census was nearly identical to the baseline census, after incorporating additional data validation measures to ensure higher data quality. Married women of reproductive age were asked about their birth history in the preceding 3 yrs during the follow-up census. However, for the purpose of this study, we limited birth history data from both the pre-implementation and follow-up censuses to within 1 year of the survey date.

#### Nested cohort

Four trained Nyaya Health Nepal community health nurses, each of whom supervises CHWs, obtained written or thumbprint consent from participants, and administered a paper-based questionnaire that was adapted from Jhpiego’s “Monitoring birth preparedness and complication readiness: tools and indicators for maternal and newborn health” [14]. The adapted tool was pilot-tested with 10 pregnant women and adjustments were made to the wording of some items for a clarity. The finalized questionnaire was administered once at the beginning of each participant’s enrollment into ANC (baseline), and then subsequently during the postpartum period (endline). Data were entered and stored using REDCap electronic data capture tools hosted at Partners Healthcare [18].

#### Key informants and focus group discussions

A staff member not involved in care delivery, and trained in qualitative interviewing, conducted the FGDs at the village clinics and the KIIs at a private location in the community. All FGDs and KIIs were audio recorded, with participant consent, and later transcribed and translated into English. Files were stored without identifiers in a private Dropbox folder.

### Statistical analysis

#### Population-level data

All outcomes – the institutional delivery rate, ANC visit completion rate, post-partum contraceptive prevalence rate, stillbirth rate, perinatal mortality rate (defined as stillbirth or neonatal i.e. < 7 days), and infant mortality rate (defined as neonatal or infant deaths < 1 year, excluding stillbirths) were binary. We summarized outcomes and mortality using frequency tables and rates per 1000 deliveries or live births (as appropriate). We truncated the two-year birth history data to one-year (from the survey date) to reduce recall bias and to prevent overlap of data from pre-implementation to follow-up. We defined an institutional delivery as one that was occurred at either Bayalpata Hospital, a village clinic, a district hospital, or a private facility. All other delivery locations (i.e. at home or on the road) were categorized as non-institutional. For women missing data on ANC visits (*n* = 10) and contraceptive use (*n* = 43) in the pre-implementation survey, we made the conservative assumption that they did not complete four or more ANC visits or use a modern method of contraception, respectively.

For each outcome, we calculated crude percentage differences of that outcome in periods of pre-implementation versus follow-up stratified by control versus intervention group. We calculated difference in differences for a crude estimate of effect of intervention. We fit logistic regression models to assess significance in intervention effect using three fixed effect parameters: indicator for pre-post period; indicator for control vs intervention village cluster; and interaction term of the two indicators i.e. pre-post*group. We determined the significance of the difference in difference estimate from the z-statistic based on maximum likelihood estimates and standard errors from the generalized linear model. We fit additional logistic models conditional on village cluster to account for potential intra-class correlation with no substantive change in significance of estimates. All statistical analyses for population-level data were conducted using R version 3.4.2 (2017-09-28) at an α = 0.05 level of significance.

#### Nested cohort data

We compared participant characteristics between the intervention and control groups at both baseline and endline using Fisher’s test for categorical variables and Wilcoxon rank sum test for continuous variables. To assess knowledge among the cohort, we calculated the proportion of all participants who correctly identified key aspects of birth preparedness, as well as key danger signs during (i) pregnancy, (ii) labor and child birth, (iii) the postpartum period, and (iv) in the newborn [14]. We used Fisher’s exact test of independence to compare these proportions between home visit care and home visit plus group ANC at endline. We also compared the change in proportions of participants meeting these criteria from baseline to endline within each group using McNemar’s test for paired categorical outcomes. All statistical analyses for cohort data were conducted using SAS version 9.3 at an α = 0.05 level of significance.

#### Qualitative data analysis

**Fig. 1:**
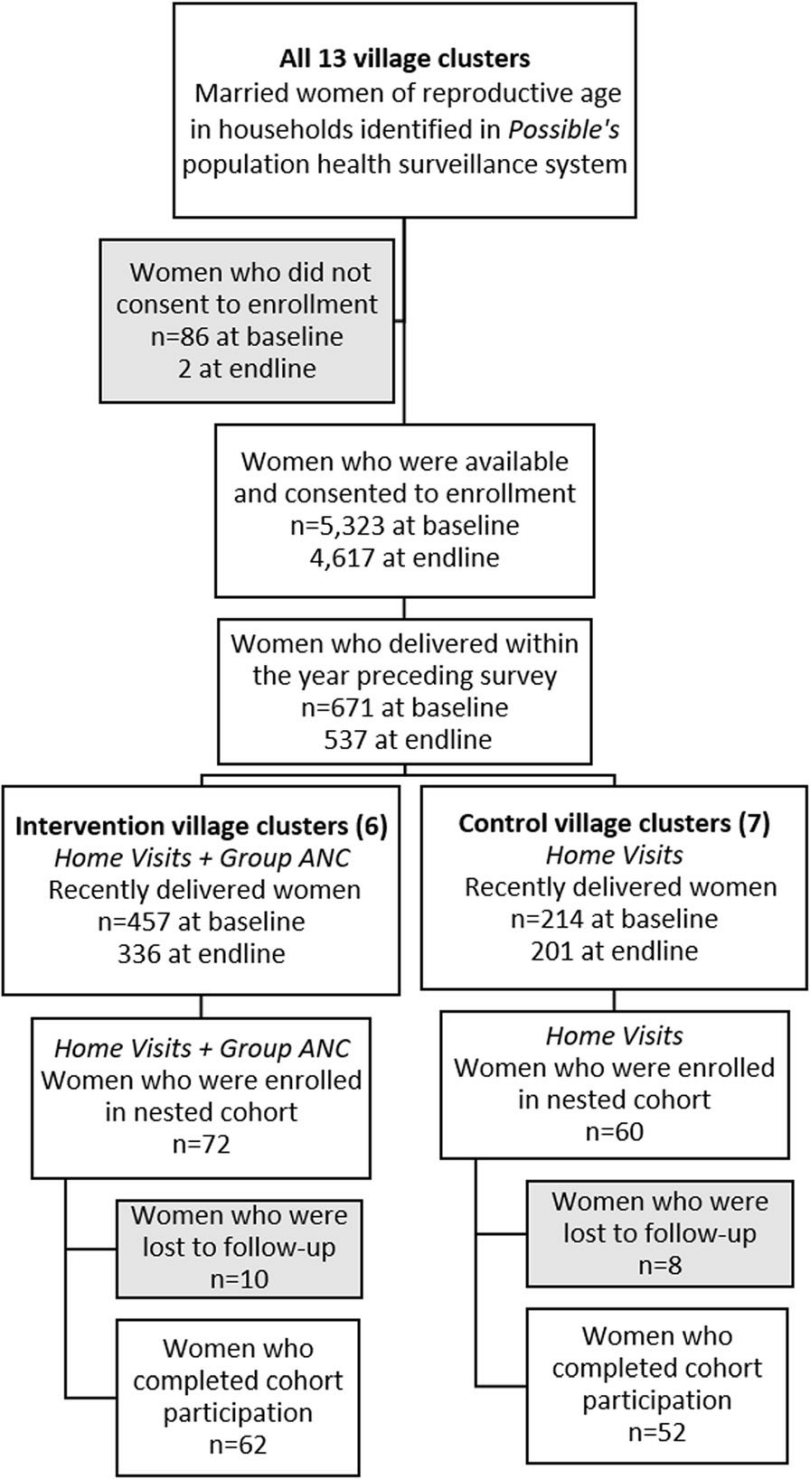
Participant enrollment flow diagram

Using a directed content analysis approach [19], two researchers read and coded each transcript independently, starting with codes derived from the theory of change, and then through open coding. The theory of change was developed during the process of intervention design and utilized literature on determinants of institutional birth and mechanisms of impact for group care (Fig. 1). The researchers initially coded a few transcripts together to ensure inter-coder consistency and then each worked independently on the remaining transcripts, developing a final codebook by comparing and combining the codes. A third expert in qualitative methods also reviewed the codebook, the themes and sub-themes, and salience of themes. The themes were then organized to interpret the results and create an analytic narrative.

## Results

### Quantitative results

All married women of reproductive age were identified via Nyaya Health Nepal’s population healthcare delivery system. Women who had a live birth within the year preceding the survey were then identified for additional survey questions assessing outcomes related to the recent birth (see Fig. 1 for participant enrollment flow diagram).

#### Population level data

The pre-implementation group consisted of 671 women – 457 from village clusters where group ANC and CHW home visit care was implemented subsequently, and 214 from village clusters with CHW home visit care and standard ANC care. The follow-up group consisted of 537 women, among whom 336 were from home visits plus group ANC village clusters and 201 were from CHW home visit care and standard ANC village clusters.

Table 1 shows the characteristics of the participants at the population level at baseline. Significant differences were seen in caste, with a higher proportion of ‘low’ caste women in the control clusters (45% vs 34%, *p* = 0.01). At the same time monthly household expenditures were significantly lower in the intervention group (Nepali Rupees 6000 vs 7000, p = 0.01). Median age of women in the intervention clusters was higher than in the control clusters (25 vs 23, *p* = 0.03), though this small difference in age is likely not clinically significant. There were no significant differences in the other measures of socio-economic position. When looking at maternal and perinatal indicators, the postpartum contraception rate and completion of ANC care were significantly higher in the control clusters compared to the intervention clusters. The institutional birth rates were not significantly different between the two groups at baseline.

**Table 1:** Characteristics of recently delivered women at baseline (*n* = 671)

	Home visits	Home visits + group care	*p*-value
*Household characteristics (n = 644)^*	(*n* = 204)	(*n* = 440)	
"Low” caste (n, %)	91 (45%)	149 (34%)	0.01*
Family size (median, IQR)	6 (4, 7)	6 (4, 7)	0.88
Monthly household expenditure in NPR (median, IQR)	7000 (5000, 10,000)	6000 (4000, 10,000)	0.01*
Land owned by family in *kattha* (median, IQR)	3 (1, 7)	3 (1, 5)	0.52
Months food secure (median, IQR)	6 (3, 12)	5 (2, 10)	0.12
*Maternal characteristics (n = 671)*	(*n* = 214)	(*n* = 457)	
Age (median, IQR)	23 (21, 27)	25 (21, 28)	0.03*
Used modern method of contraception (n, %)	54 (25%)	72 (16%)	< 0.01*
Institutional births (n, %)	171 (80%)	371 (81%)	0.75
Completed 4 ANC visits (n, %)	193 (90%)	363 (79%)	< 0.01*

*^Note: 10 households (4.6%) in the home visit care only clusters and 17 households (3.7%) in the home visit plus group care clusters were missing data on caste, family size, land owned, months food secure and household monthly expenditures, and were excluded from the above analyses, thus leaving an effective sample size of 644. Twins are considered a single delivery. For the 4 ANC visit completion rate and post-partum contraceptive rate metrics, missing observations were assumed to have not completed all 4 ANC visits and not be using a modern method of contraception respectively*

*Statistically significant

Comparing the combined baseline data of both intervention and control clusters with the endline data, there was a significant improvement in primary outcomes of institutional birth rate (81% vs 93%, *p* < 0.001), ANC completion rate (83% vs 90%, *p* = 0.001), and PPCPR (19% vs 47%, p < 0.001). However, the difference in difference analysis from baseline to endline showed no significant comparative difference between intervention and control clusters (Table 2).

**Table 2:** Primary outcomes at baseline and endline: difference in difference analysis^

	Baseline			Endline			Difference	
*Outcomes*	*Control*	*Intervention*	*Diff*	*Control*	*Intervention*	*Diff*	*Diff-Diff*	*p-value*
Institutional birth rate	79.5%	81.2%	1.7%	94.0%	91.5%	−2.5%	−4.2%	0.25
ANC (4+)	89.8%	80.3%	−9.5%	93.0%	88.8%	−4.2%	5.3%	0.55
Post-partum contraceptive prevalence rate	25.1%	16.2%	−8.9%	59.8%	38.0%	−21.8%	−12.9%	0.22
Stillbirth rate*	4.6	19.3	14.7	19.7	26.6	6.9	−7.8	0.35
Perinatal mortality rate*	23.1	30.0	6.9	24.6	38.5	13.9	7.0	0.80
Infant mortality + Stillbirth rate*	23.1	38.6	15.5	29.6	41.4	11.8	−3.7	0.80
Infant mortality rate*	18.5	19.3	0.8	9.9	14.8	4.9	4.1	0.72

*Results are reported per 1000 total births (= live births + stillbirths)

*^Note: the unit of analysis for the difference in difference analysis is every birth, and not every woman. There are 11 pairs of twins in the baseline and 4 pairs of twins in the endline*

#### Nested cohort results

During the first 3 months of the study, we enrolled 72 and 60 women for the nested cohort in the intervention and control clusters, respectively. No women who were approached for enrollment into the cohort declined participation. At the follow up visit after the completion of the pregnancy, 18 (13.6%) participants did not complete the endline survey and were excluded from the cohort data analysis – among these, 10 (13.9%) belonged to the intervention group and eight (13.3%) to the control group. Sixty-two women from intervention clusters and 52 from control clusters remained in the cohort and had completed both baseline and endline surveys. The cohorts had no significant demographic (age, caste, parity, family size) and socio-economic (education, household expenditure, subsistence, land) differences.

Surveys of knowledge related to danger signs revealed low scores in both intervention and control clusters at baseline (Table 3). Knowledge of key danger signs during pregnancy significantly improved from baseline to endline in the intervention clusters (2 to 31%, p < 0.001). Knowledge of key danger signs related to labor and childbirth, the postpartum period, and the newborn did not significantly improve in the intervention clusters. In the control clusters there was no significant improvement in knowledge for any of the key danger signs. Birth preparedness knowledge decreased in both intervention and control clusters.

**Table 3:** Knowledge of danger signs and birth preparedness in nested cohort

*Knowledge indicators at end line by intervention clusters, n = 114*			
Participants who identified	Intervention cohort (*n* = 62)	Control cohort (*n* = 52)	p-value
Key danger signs during pregnancy	19 (31%)	5 (10%)	0.01
Key danger signs during labor and childbirth	6 (10%)	4 (8%)	0.75
Key danger signs during the postpartum period	7 (11%)	5 (10%)	1.00
Key danger signs in the newborn	2 (3%)	0 (0%)	0.50
Key aspects of birth preparedness	18 (29%)	14 (27%)	0.84
*Change in knowledge indicators within intervention clusters, n = 62*			
Participants who identified	Baseline	Endline	*p*-value
Key danger signs during pregnancy	1 (2%)	19 (31%)	< 0.001
Key danger signs during labor and childbirth	1 (2%)	6 (10%)	0.06
Key danger signs during the postpartum period	3 (5%)	7 (11%)	0.21
Key danger signs in the newborn	1 (2%)	2 (3%)	0.56
Key aspects of birth preparedness	25 (40%)	18 (29%)	0.16
*Change in knowledge indicators within control clusters n = 52*			
Participants who identified	Baseline	Endline	*p*-value
Key danger signs during pregnancy	1 (2%)	5 (10%)	0.10
Key danger signs during labor and childbirth	0 (0%)	4 (8%)	^
Key danger signs during the postpartum period	2 (4%)	5 (10%)	0.08
Key danger signs in the newborn	0 (0%)	0 (0%)	^
Key aspects of birth preparedness	22 (42%)	14 (27%)	0.05

*^ No statistics computed since no participants at baseline and/or end line identified signs*

When comparing intervention and control cohorts at endline, the intervention cohort had a significantly larger proportion of participants who were able to identify key danger signs during pregnancy. There was no significant difference between the intervention and control cohorts for knowledge of other key danger signs or birth preparedness.

There was no significant difference in ANC visit completion between intervention and control cohorts at endline, which is consistent with the population level data (Table 4). There was also no significant difference between intervention and control cohorts on birth planning practices as reported for their recent pregnancy.

**Table 4:** ANC and birth planning practices among participants at endline

Category	Outcome	Intervention group (n = 62)	Control group (n = 52)	p-value
*ANC practices*	Attended at least 4 ANC visits during pregnancy	56 (90%)	50 (96%)	0.29
*Birth planning practices*	Planned for delivery by skilled birth attendant	22 (35%)	15 (29%)	0.55
	Arranged transport to hospital	34 (55%)	29 (56%)	1
	Saved money	58 (94%)	50 (96%)	0.69
	Arranged food	58 (94%)	46 (88%)	0.51
	Arrange clothes	60 (97%)	47 (90%)	0.24
	Identified a birth companion	44 (71%)	32 (62%)	0.32
	Identified a blood donor	16 (26%)	12 (23%)	0.83
	Made multiple preparations^	13 (21%)	8 (15%)	0.48

*^ Planned for delivery by skilled birth attendant, arranged transport to hospital, and saved money*

Patient satisfaction with ANC care is shown in Table 5. The majority of both intervention and control cohorts found ANC sessions very useful (92, 94%, *p* = 0.73). Additionally, both intervention and control cohorts reported that ANC providers provided excellent care (94, 90%, p = 0.73). A significantly larger proportion of the intervention cohort found ANC care to be “very enjoyable” (84% vs 60%, *p* = 0.01).

**Table 5:** Patient satisfaction with ANC sessions at endline

Category	Intervention group (n = 62)	Control group (n = 52)	p-value
*Quality of ANC sessions*			
Not useful	0 (0%)	0 (0%)	0.73
Somewhat useful	5 (8%)	3 (6%)	
Very useful	57 (92%)	49 (94%)	
*Quality of ANC provider*			
Provided poor care	0 (0%)	0 (0%)	0.73
Provided mediocre care	4 (6%)	5 (10%)	
Provided excellent care	58 (94%)	47 (90%)	
*ANC level of enjoyment*			
Not enjoyable	0 (0%)	0 (0%)	0.01
Somewhat enjoyable	10 (16%)	21 (40%)	
Very enjoyable	52 (84%)	31 (60%)	

### Qualitative results

We conducted 10 semi-structured interviews with the following key informants: group participants (2), providers (2 CHW group facilitators, 2 government care-providers), community leaders (2), and Nyaya Health community health supervisory staff (2). Two KIIs were excluded from analysis due to poor quality of the interview, translation, and transcription. The excluded interviews included one government care provider and one community leader. A FGD was conducted in each of the six intervention village clusters. In total, we analyzed eight KIIs and six FGDs.

The results are reported in three categories: those that support or relate to the theory of change, those that emerged newly from the qualitative data, and those that addressed acceptability and implementation barriers of the intervention. The source of the exemplary quotes provided below is included after each quote. Themes or codes that were identified multiple times are marked for saliency with “n” indicating the number of times mentioned in entire set of qualitative data.

#### Results supporting or related to the theory of change

**Fig. 2:**
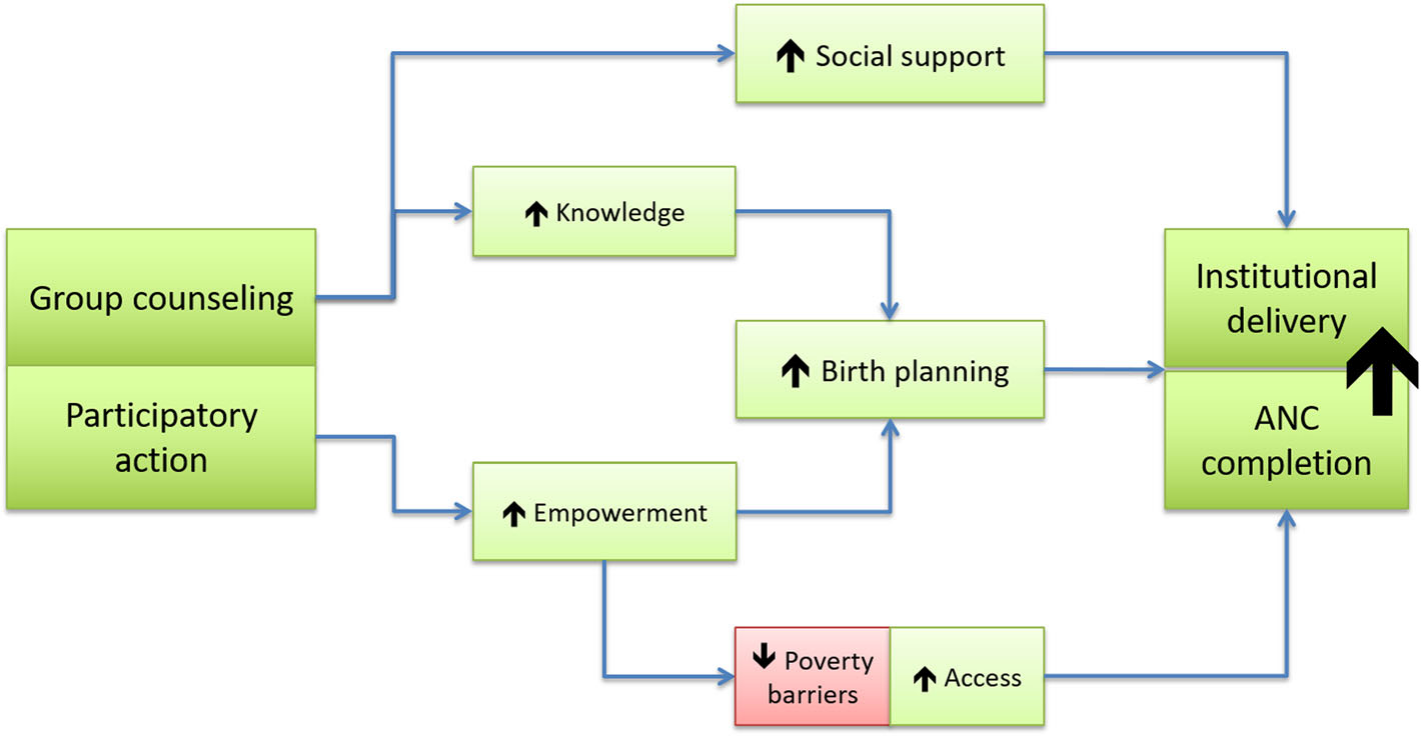
Group ANC theory of change

Several themes that emerged from our qualitative analysis supported our theory of change (Fig. 2): increased participant knowledge, addressing barriers to institutional birth, increased social support, and increased social empowerment. The specific topics of knowledge that women discussed included: institutional delivery and facilities, nutrition, pregnancy care, self-care, danger signs, hygiene, infant care, and birth planning. One woman stated, “We wouldn’t have known or learned a lot staying at home. We got to learn and understand a lot of things” (FGD3). The theme of social empowerment was manifested in being able to speak up in a group setting, as one woman stated, “Before no matter how educated or uneducated we were, we used to feel uncomfortable and shy to talk with people or in front of people. Now, after coming to these sessions and participating in discussions, we have learned to talk as well. Above all else, we have learned to talk and feel comfortable expressing ourselves now” (FGD 1).

In the theory of change, group ANC was anticipated to increase birth planning, however data revealed several challenges. A number of participants noted the challenges of saving money for birth and finding a blood donor (*n* = 12). In referring to birth planning one CHW stated, “When we fill (the birth planning form), it is problematic … some pregnant women won’t even have money to eat and that same woman’s neighbor will have a few lakhs [approximately US$1000s] in her bank account … To such people to tell them, I feel a bit awkward to ask them, (but for) others there is not such a problem” (KII CHW1). A group participant similarly noted, “Money is also difficult. You have to use all your resources to arrange it [transportation]. For those who have businesses/work it might be easy, but for people like us, it is difficult” (FGD 3).

#### Results of emergent themes

The following themes were identified in the data and not directly related to the a priori theory of change. Participants saw group ANC as a platform for discussion, learning from each other and sharing (*n* = 13). One FGD participant noted, “We came together as a group, discussed things … We learned things we did not know before by listening to people who did” (FGD 4). Participants appreciated and asked for more incentives for attending group ANC (*n* = 20). Incentives noted by participants included the government financial incentive for complete ANC, ultrasound, and free services closer to home. Participants recognized the direct promotion of institutional birth in group ANC (*n* = 29).

Providers interviewed noted that women seemed to enjoy group ANC (*n* = 7). A CHW stated,

Now due to this program pregnant women are also enjoying it a lot. Now pregnant women come and ask us, ‘When are we coming for our next checkup? When are we going next?’ They ask this and then when they get to sit in a group … Now they don’t have the ‘*aa*, why do we need to go for checkup?’ kind of mentality (KII CHW 1).

Providers noted an improvement in participation and acceptance of group ANC over time. They expressed that conducting group ANC was easy (*n* = 4) and stressed the importance of using guides and having ongoing training. Additionally, providers noted facilitation was enjoyable (n = 4). One facilitator emphasized the utility of ultrasound during group ANC. Providers noted that group ANC increased the acceptance of and attendance at ANC and PNC (n = 7). They also noted a positive effect on family planning, namely an increase in planning for and utilization of contraception (*n* = 6), and a positive effect on maternal and child outcomes through referrals (n = 6). One provider, a government village clinic staff, noted difficulties inherent to the village clinic that made group ANC challenging (*n* = 3). Providers and a community leader discussed the collaboration of the organization, Nyaya Health Nepal, and the government clinics as being positive for improving services and reaching targets. Providers discussed the CHW home visits as important for identifying and engaging pregnant women as well as building trust (*n* = 4).

Nyaya Health Nepal staff members noted several ways in which group ANC has helped both women (*n* = 12) and care delivery at the village health clinics (n = 4), including improving confidence to give birth in the hospital, nutrition, decentralized services, detection of high-risk pregnancies, and creating efficiency and learning opportunities for the providers. A community health nurse said,

The things they (pregnant women) didn’t understand, now they have the opportunity to understand … They’ve gotten to do lab tests and USGs (ultrasounds), that’s very good. Right from the start we identify high risk pregnancies and complications are not there. Women used to die, they would deliver at homes, such things have reduced a lot. (KII community health nurse).

One staff member in a supervisory role noted increased confidence and the openness among CHWs as they developed their identity as leaders in the community. At the same time, another staff member noted how variable buy-in to the program from government staff affected care delivery. Staff discussed other challenges, such as equipment issues and increased work burden on the government midwives. Staff and community leaders discussed the opportunity they saw in scaling the intervention: to improve access to services for women and other benefits of the program (*n* = 5). They also expressed concerns about scaling the intervention given the staff requirements and need for government buy-in (n = 4).

#### Results related to acceptability and implementation barriers

We asked questions to all groups of participants to examine acceptability and implementation barriers. The responses revealed that participants saw the benefits of group ANC over individual care (*n* = 20), such as more counseling and learning, a closer relationship with the clinic staff, and increased services closer to home. One woman stated, in referring to the clinic staff, “We know them better now. Before we would see them around and not know them. If they called us for medication or something, we would come, that was it. But, now we know them better, feel comfortable with them” (FGD 2). There was also a perception of improved facilities and care at all levels: home, village clinic, and hospital (*n* = 15).

Challenges that participants raised included groups taking too much time out of their busy lives, being made to feel ignorant in groups or unable to speak well, and too much information with poor retention of the knowledge. When asked about recommendations for improvement, participants suggested further increasing services at the village clinic, specifically related to ultrasound. Providers noted that providing group care was easier than individual care, that acceptance of institutional birth seemed greater, that women seemed to enjoy group care, and that the women gained social support. They noted that women often could not come on time to groups, issues with the birth planning tool (a form used by the CHWs to document each woman’s birth plan), and challenges with birth planning itself due to poverty.

## Discussion

Our quantitative results reveal that the group ANC intervention did not lead to an improvement in the outcomes of institutional birth, ANC care completion, or contraceptive prevalence over the home visit care only clusters. We did observe a significant improvement in all three of these outcomes in the overall catchment area population from pre-implementation to follow-up. We hypothesize that the CHW home visit care program has likely driven the improvement in these outcomes, though it is possible that there are other unmeasured reasons for this change over time. Given the short time-period and the relatively stable environment, it is likely that the CHW home visit care program is responsible for the changes observed [20].

The nested cohort analysis reveals some success in group ANC at increasing knowledge of pregnancy danger signs, which is consistent with knowledge increases seen in group ANC implemented in Ghana [21]. However, in our program no relative success was observed in other key knowledge areas. The success for pregnancy danger signs may be due to the relatively high frequency that this topic was discussed compared to other topics during the course of group ANC, as described in the complementary paper [11]. All of the knowledge indicators were quite low at the baseline and the endline in both clusters of the study, indicating very poor health literacy in this community and that neither the home visit care nor the group ANC program were particularly successful in increasing knowledge based on international standards [22].

Knowledge of key aspects of birth preparedness actually decreased in both intervention and control clusters. This result is difficult to explain given that the survey questions were the same at baseline and endline, and counseling materials for both home care visits by CHWs and group ANC addressed birth preparedness. Our counseling methods in both clusters need to be examined closely and improved upon, perhaps adapting to the very low health literacy of this population, given how far we are from achieving an acceptable knowledge level among women on key danger signs and birth planning.

Our qualitative analysis reveals that women found that the groups provided an opportunity for learning and discussion, learning from each other, and a source of social support and empowerment. This is consistent with findings from a qualitative analysis of the Centering Pregnancy model [23]. They also reported perceived improvement and increase in availability of services at the village clinic—specifically mentioning labs, ultrasound, and counseling. Improved decentralized services is a unique aspect of our Group ANC program, not described in Centering Pregnancy or other group ANC models in low and middle-income countries [24, 25]. Providers noted that groups made delivering ANC services easier, led to improved acceptance of ANC, facility-based delivery services, and family planning methods, and provided a space for women’s empowerment and social support. Provider perspectives are consistent with qualitative data from group ANC piloted in India [26]. Providers also noted the importance of home visits as part of the intervention and the value of collaboration between the nurse midwives and the CHWs. Finally, providers noted that village clinic services were improved and reported the importance of expanding the intervention. The analysis also revealed several challenges to the implementation and the effectiveness of the program. Both participants and providers noted difficulties with birth planning in a place where transportation, social support, and poverty remain challenges for women and their families.

The qualitative data reveal the acceptability and some implementation barriers of the intervention. Our qualitative data support our quantitative findings that group care is enjoyable for participants (and providers as well), and that there is some educational benefit. The challenges, particularly the socioeconomic ones, described with birth planning and the importance of home visits may explain why we did not see a significant difference in our primary outcomes. What remains challenging to reconcile is the perception that group ANC improved services and as a result, outcomes, while our quantitative data show improvement that is similar across control and intervention village clusters. Additionally, the qualitative data show women felt that the group intervention promoted learning and increased knowledge in many subject areas, yet the quantitative improvement in knowledge scores was limited to pregnancy danger signs in the nested cohort analysis. Of note, qualitative data were only collected in intervention areas and so are not representative of the whole cohort.

There are several limitations to the quantitative data. Our study was designed to enroll 2400 participants to achieve 80% power to detect effect size 5%. Because our enrollment was lower than expected, our analysis is underpowered. We did not have 80% power to detect an effect size of 5% as expected. Additionally, the intervention was evaluated over a very short time frame. The recommended time frame to look at pregnancy-related interventions is three to five years given that a pregnancy is 10 months long [14]. We made the decision to evaluate the intervention after 1 year due to practical and political considerations, despite the likely bias towards a null result in a very short timeframe. The population-level data are also limited by differences in the timing and length of the survey periods. We tried to keep the length of the survey periods the same before and after the implementation of the intervention to minimize differences in potential sample size, however changes in logistical methods to improve exhaustive sampling required additional time in the follow-up census survey frame. Finally, given the small sample size, and a programmatic and political allocation of control versus intervention groups, there were likely significant differences between the clusters themselves. In particular, the intervention group tended to be larger, with poorer road access, farther from the hospital, and with greater distances between village clinics and households. This likely further tilted the data in the direction of the null hypothesis.

While the study was designed to focus primarily on the effectiveness of the group care intervention, the two arms of the study both included new programs: the CHW home care visits and the home care visits integrated with group ANC visits. Given the CHW home visits program was also new, there was likely ongoing change and iteration during the study period leading to differing exposures to the participants. All study data were collected by unblinded Nyaya Health Nepal staff exposing the study to potential observer bias. However, both arms included new programs that were being evaluated which likely mitigated this bias. Information bias, in which patients give attenuated responses because of the dynamic between providers and patients, is also likely.

The population level data were likely affected by recall bias as the survey questions were designed to ask about the most recent birth within 1 year of survey. The one-year time limitation was selected to minimize this bias. In the population level data collected after 1 year of implementation, the exposure to interventions remains unclear, as we did not assess the number of CHW home visits or group ANC visits for every woman at the population level. We aimed for complete penetration of the interventions throughout the population, however we did not specifically assess penetration at endline. Given that the intervention enrollment was linked to active pregnancy case finding at the household level, it is likely that we achieved close to complete penetration. However, given the timing of the surveys and assuming an equal distribution of births throughout the year, we estimate ~ 75% of births in the population-level data would have received the refined group ANC intervention, since most (if not all) women who delivered between March and May 2015 (i.e. the first 3 months of the intervention) would have been > 28 weeks pregnant at the time of enrollment into group ANC.

## Conclusions

In the current literature, though there are a few studies of group ANC in low-resource and rural settings, there is clearly a gap for additional evidence [25]. This study examines a group model of ANC based out of a rural village clinic, and lead by CHWs and government midwives. We presented data here that indicate that group ANC did not have an impact on the institutional birth rate nor antenatal care completion, with the limitations of low power and the short time frame of the study. We also show that group ANC led to improved participant satisfaction and a modest impact on knowledge.

Based on the qualitative data, we see the importance of strengthening village clinic services, specifically in decentralizing laboratory and ultrasound services and building stronger ties between the community and clinic providers. Additionally, the qualitative data illustrate the value of the group setting for discussion, knowledge sharing, empowerment, and social support. These things are not possible to achieve with CHW home visits alone. Given the improvement in outcomes of the combined intervention of CHW home visits and group care, specifically an achievement of 93% institutional birth, we see the value of studying this combined intervention further in a larger population and over a longer time frame.

## Data Availability

De-identified quantitative data are available on request by emailing: research@possiblehealth.org and will be posted in a publicly-accessible data repository. Full transcripts of qualitative data are not available as they contain quotes and identifiable information that could compromise the identify of participants.

## Abbreviations

ANC: Antenatal care
CHW: Community health worker
FGD: Focus group discussion
KII: Key-informant interview
PPCPR: Post-partum contraceptive prevalence rate
